# Assessing the Intraoperative Accuracy of Pedicle Screw Placement by Using a Bone-Mounted Miniature Robot System through Secondary Registration

**DOI:** 10.1371/journal.pone.0153235

**Published:** 2016-04-07

**Authors:** Keng-Liang Kuo, Yu-Feng Su, Chieh-Hsin Wu, Cheng-Yu Tsai, Chih-Hui Chang, Chih-Lung Lin, Tai-Hsin Tsai

**Affiliations:** 1 Graduate Institute of Clinical Medicine, College of Medicine, Kaohsiung Medical University, Kaohsiung, Taiwan; 2 Graduate Institute of Medicine, College of Medicine, Kaohsiung Medical University, Kaohsiung, Taiwan; 3 Department of Neurosurgery, Kaohsiung Medical University Hospital, Kaohsiung, Taiwan; University of Michigan, UNITED STATES

## Abstract

**Introduction:**

Pedicle screws are commonly employed to restore spinal stability and correct deformities. The Renaissance robotic system was developed to improve the accuracy of pedicle screw placement.

**Purpose:**

In this study, we developed an intraoperative classification system for evaluating the accuracy of pedicle screw placements through secondary registration. Furthermore, we evaluated the benefits of using the Renaissance robotic system in pedicle screw placement and postoperative evaluations. Finally, we examined the factors affecting the accuracy of pedicle screw implantation.

**Results:**

Through use of the Renaissance robotic system, the accuracy of Kirschner-wire (K-wire) placements deviating <3 mm from the planned trajectory was determined to be 98.74%. According to our classification system, the robot-guided pedicle screw implantation attained an accuracy of 94.00% before repositioning and 98.74% after repositioning. However, the malposition rate before repositioning was 5.99%; among these placements, 4.73% were immediately repositioned using the robot system and 1.26% were manually repositioned after a failed robot repositioning attempt. Most K-wire entry points deviated caudally and laterally.

**Conclusion:**

The Renaissance robotic system offers high accuracy in pedicle screw placement. Secondary registration improves the accuracy through increasing the precision of the positioning; moreover, intraoperative evaluation enables immediate repositioning. Furthermore, the K-wire tends to deviate caudally and laterally from the entry point because of skiving, which is characteristic of robot-assisted pedicle screw placement.

## Introduction

Pedicle screw placement is widely used in restoring spinal stability and correcting deformities [1]. The accurate placement of pedicle screws in patients with scoliosis, reoperation, osteoporosis, or anomalous congenital spinal developmental is extremely challenging [2–5]. Various surgical techniques (e.g., image-guided surgery and robot-assisted pedicle screw placement) and devices (e.g., navigation devices) have been developed to improve the accuracy of pedicle screw placements [6, 7].

The Renaissance robotic system (Renaissance, Mazor Robotics Ltd., Israel) guides surgeons in implanting pedicle screws. Clinical studies have suggested that the accuracy of pedicle screw placement guided by the Renaissance robotic system is higher than that of manual or image-guided operations; however, the accuracy is not as consistent as that achieved using navigation devices [8, 9].

In patients who do not undergo postoperative computerized tomography (CT) (this is not a routine procedure in many hospitals), the accuracy of pedicle screw placement can be verified through X-ray images alone, and the accuracy of the evaluation depends solely on the operator’s experience and the clinical outcome [10, 11]. However, through use of the Renaissance system, the accuracy of screw placements can be more precisely verified immediately after secondary registration by comparing the planned trajectory and the Kirschner wire (K-wire) placement.

In the present study, we developed an intraoperative classification system of pedicle screw placement accuracy through secondary registration. Furthermore, we evaluated the benefits of the Renaissance robotic system in pedicle screw placement, particularly after secondary registration, and examined the factors affecting pedicle screw placement accuracy.

## Materials and Methods

### Inclusion and exclusion criteria

We retrospectively analyzed patients with both degenerative and isthmic lumbar spondylolisthesis who either underwent or did not undergo transforaminal lumbar interbody fusion (TLIF) at Kaohsiung Medical University Hospital between January and March 2015. Our surgical indications included conservative treatment failure, ongoing neurological deficit, intractable back pain, and deformity progression. We included patients who received a diagnosis of lumbar spondylolisthesis, were refractory to medical treatment for 6 months, and underwent correction through robot-assisted transpedicular screw fixation. We excluded those who had spinal malignancy, a history of spinal trauma or fracture, spinal infection, or osteoporosis. In total, we recruited 64 patients.

### Ethics statement

This clinical study was approved by the Institutional Review Board of Kaohsiung Medical University Hospital: (No: KMUHIRB-E(I)-20150167). Written informed consent was obtained from all the participants. Before the analysis, patient data were de-identified and anonymously analyzed.

### Clinical characteristics

We enrolled 64 patients (14 men and 50 women) with both degenerative and isthmic lumbar spondylolisthesis who underwent robot-assisted spine surgery at Kaohsiung Medical University Hospital (Kaohsiung, Taiwan) between January and March 2015. After the patients’ records were de-identified, we obtained the following perioperative clinical parameters: age, sex, osteoporosis data, and information on previous operations, comorbidities, operative times, blood loss, and X-ray exposure (Table 1). All the patients percutaneously underwent transpedicular screw fixation, with or without TLIF. In total, 317 pedicle screws were implanted in the 64 patients. The mean age of the patients was 65.56 ± 13.31 years, the mean body mass index (BMI) was 25.58 ± 4.66 kg/m^2^, the mean operative time for robot-assisted K-wire implantation was 38.47 ± 14.98 minutes, and the mean number of intraoperative X-ray films was 6.33 ± 2.85 spot films (single exposures recorded as radiographic images). The size of the pedicle screws varied. The size was determined according to the diameter of the pedicles on the preoperative CT scans; this approach was adopted to avoid breaching the pedicles and to decrease the possibility of skiving from the facet joint.

**Table 1:** Clinical characteristics of the 64 patients who underwent robot-guided pedicle screw placement.

**Numbers**	**64**
**Age (years)**	**65.56±13.31 (20–89 y/o)**
**Gender(M/F)**	**14/50**
**BMI**	**25.58±4.66**
**No of pedicle screws**	**317**
**Op time(min)**	**38.47±14.98**
**Intraoperative X-ray (spot film)**	**6.33±2.85**

### Robotic surgical techniques

The Renaissance robotic system was used as follows [7, 12–15]:

1. **Preoperative planning**: Spiral CT images (1-mm intervals) were converted to three-dimensional reconstruction images to plan the optimal trajectory, screw diameter, and length of the instruments used in the surgery.
2. **Attachment to the patient’s spine**: An appropriate mounting frame was attached to the patient’s spine.
3. **Registration**: The Renaissance robotic system automatically registered the intraoperative images with the preoperative CT images by using two radiographic images (anteroposterior and oblique views).
4. **Robot packaging and initiation**: The robot was attached to the mounting frame to ensure that it could be positioned according to the preoperative plan.
5. **Drilling implementation**: A guiding tube was set toward the pedicle, and drilling was subsequently performed by a surgeon. A K-wire was then inserted using the robot.
6. **Secondary registration (Reregistration)**: After the K-wire implantation was completed, anteroposterior and oblique fluoroscopic images were obtained and automatically registered with the preoperative CT images.

### Secondary registration

**Fig 1:**
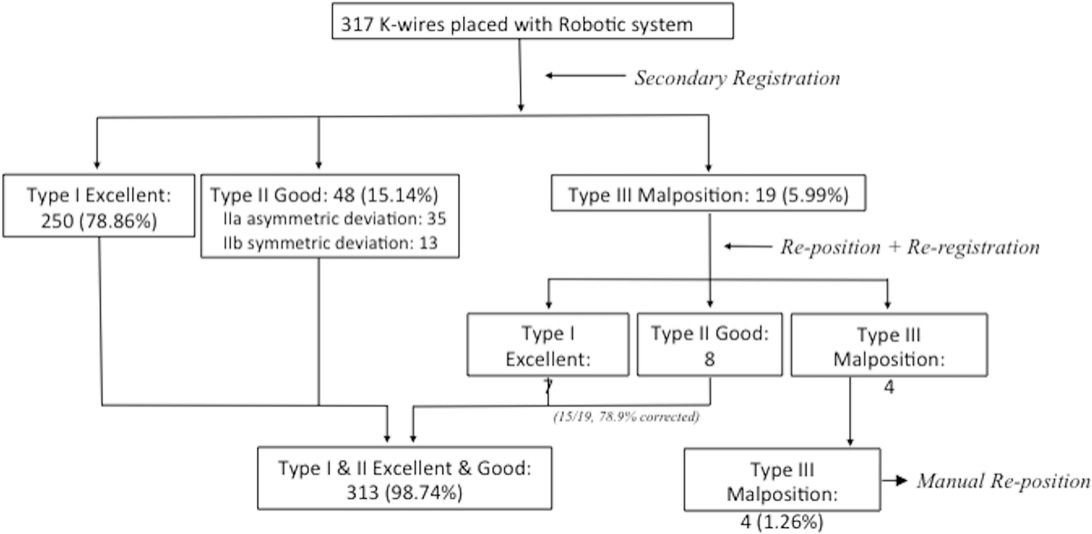
Process of the secondary registration and repositioning of the K-wire

Two additional X-ray images were obtained and registered with the preoperative CT images. This step, called secondary registration or *reregistration*, was used to compare the post- and preoperative anterioposterior and oblique fluoroscopic images. Reregistration was performed after K-wire implantation rather than after transpedicular screw implantation, because the robot cannot determine the tract of the transpedicular screws from the trajectory because the screws cause severe interference. The K-wire placement accuracy was determined through reregistration. When the K-wire deviated >3 mm from the planned trajectory, repositioning was performed. Because the K-wire and planned trajectory could be simultaneously evaluated by the robot, reregistration was repeated after repositioning to ensure that the K-wire tract was within 3 mm of the planned trajectory. The purpose of reregistration was to assist the operator in estimating the deviation of the K-wire from the planned trajectory, to verify whether the K-wire was malpositioned immediately during the operation, and to avoid performing a second revision after the first operation. All the processes of the secondary registration and repositioning of the K-wire are summarized in Fig 1.

Among the 19 K-wire implantations initially classified as malpositioned, 15 were improved to “excellent” and “good” categories (i.e., type IIIa classification). However, the remaining 4 malpositioned K-wires requirement manually adjustment (i.e., type IIIb classification).

### Postoperative status

#### Accuracy of intraoperative K-wire placement

**Fig 2:**
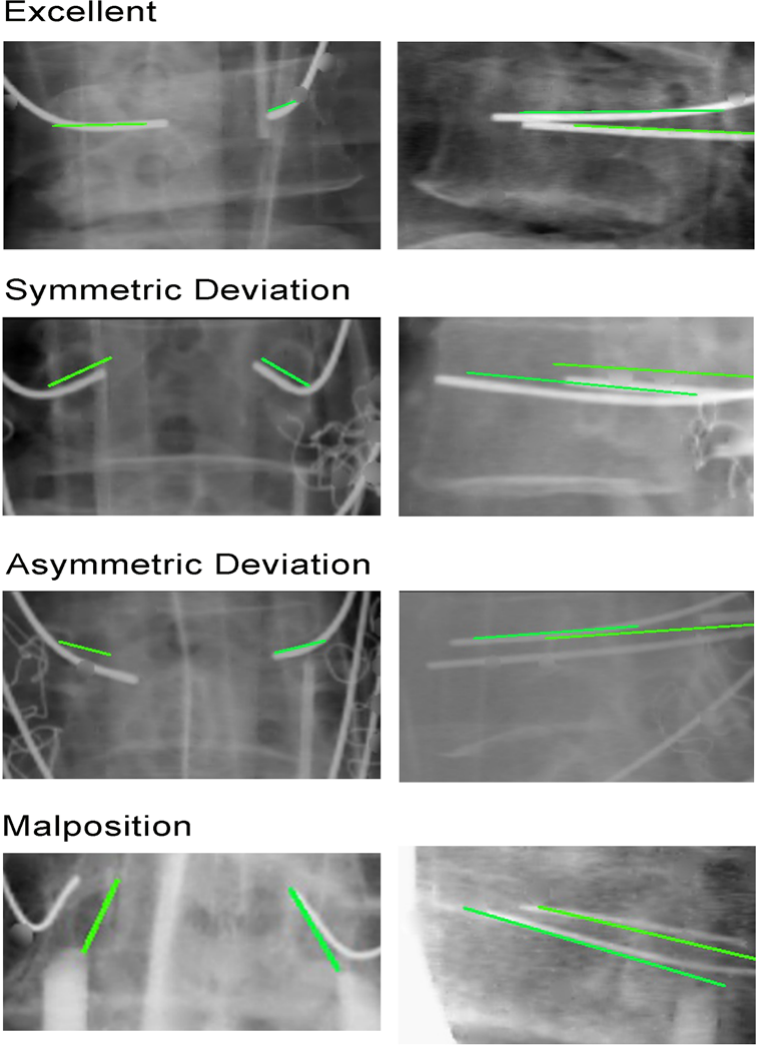
**Classification system for assessing intraoperative accuracy** Fig 2A depicts type I classification, Fig 2B and 2C depict type II classifications, and Fig 2D depicts Type III calssification. (green line: planned trajectory; white line: implanted K-wire).

By using secondary registration, we developed a classification system (Fig 2) to verify the accuracy of the K-wire placement relative to the planned trajectory. K-wire placements were classified into the following three types: type I (excellent, <1-mm deviation from the planned trajectory), type II (good, 1–3-mm deviation from the planned trajectory), and type III (malpositioned, deviation > 50% of the screw diameter; i.e., >3 mm). We subcategorized the type II cases into type IIa (asymmetric deviation), in which one pedicle screw follows the planned trajectory and the other pedicle screw, which is at the same level, does not; and type IIb (symmetric deviation), in which both pedicle screws do not follow the planned trajectory. Similarly, we subcategorized the type III cases into type IIIa (malpositioned, robot-repositionable), in which the screws can be repositioned using the Renaissance robotic system; and type IIIb (malpositioned, robot-nonrepositionable), in which the robot cannot reach the planned trajectory because of design limitations or several failed repositioning attempts (manual implantation is necessary in these cases).

### K-wire entry point

**Fig 3:**
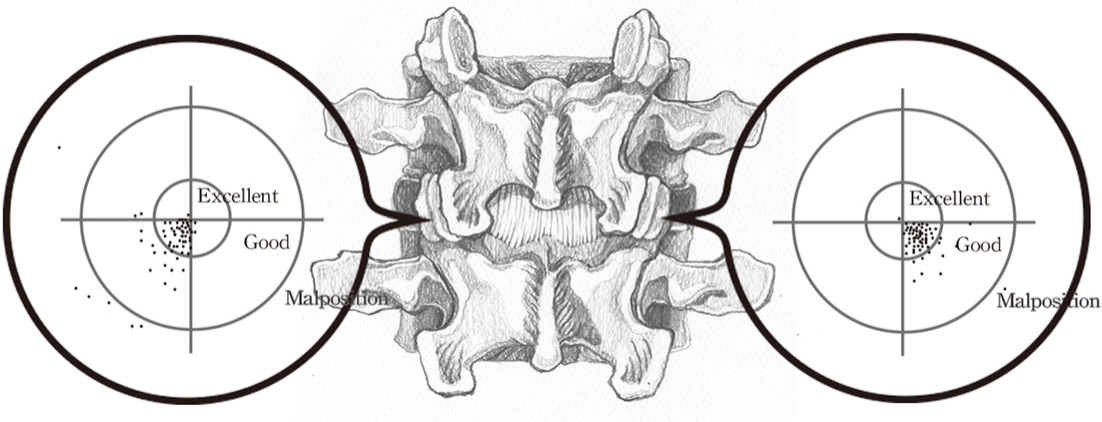
Entry point of the K-wire. Preoperative planned entry point is the center of the circle. Most K-wire entry points deviated caudally and laterally.

Fig 3 depicts the distribution of the K-wire entry points. The preoperative planned entry point was situated at the center of the circle (zero). We calculated the deviation of the K-wire entry point from the planned entry point to determine whether the screws deviated laterally, medially, caudally, or cranially (Fig 3).

### Postoperative accuracy of pedicle screw placement

The accuracy of pedicle screw implantations has been classified into the following four categories [16,18] through postoperative biplanar conventional X-ray images before hospital discharge: grade I, adequate accuracy and safety with guidance from the Renaissance robotic system; grade II, pedicle screw malpositioned; grade III, pedicle screw placement performed manually; and grade IV, operation not performed as planned preoperatively.

### Statistical analysis

All parametric data are expressed as mean ± standard deviation. The accuracy before and after repositioning and secondary registration was compared using the goodness of fit test. (SPSS Version 19.0 for Windows, IBM, NY, USA.) A P value of <0.05 was considered statistically significant.

## Results

### Accuracy of K-wire placement

Table 2 presents the accuracy of the K-wire placements investigated in this study. Types I, II, and III accounted for 78.86%, 15.14%, and 5.99% of the placements, respectively. Of the 15.14% type II placements, 11.04% were type IIa (asymmetric deviation) and 4.10% were type IIb (symmetric deviation). Of the 5.99% type III placements, 4.73% were type IIIa (robot-repositionable) and 1.26% were type IIIb (robot-nonrepositionable). Entry points in the type IIIa status cases attained type I (from 78.86% to 81.07%) and type II (from 15.14% to 17.67%) status before and after repositioning, respectively. The accuracy of the robot-guided K-wire implantations was 94.00% and 98.74% (inclusive of types I and II) before and after repositioning, respectively. A significant difference was identified with the goodness of fit test (p < 0.0001; Table 2).

**Table 2:** Classification of the accuracy of the K-wire placement through the secondary registration.

	Accuracy (n/%)			
	Before reposition	After secondary registration & reposition		P value
**Accuracy Types**				<0.0001 *
**Type I Excellent**	**250/78.87%**	**257/81.07%**		
**Type II Good**	**48/15.14%**	**56/17.67%**		
**IIa Asymmetric Deviation**	**35/11.04%**	**41/12.94%**		
**IIb Symmetric Deviation**	**13/4.10%**	**15/4.73%**		
**Type III malposition**	**19/5.99%**	**4/1.26%**		

^a^ p value was calculated using the goodness of fit test

* p value <0.05

### K-wire entry point

Most of the K-wire entry points deviated caudally and laterally. Neither screw-related complications nor permanent neurological deficits were observed during the follow-up period. Therefore, secondary revision surgery was not required.

### Postoperative accuracy of pedicle screw placement

Based on Hu’s classification [16,18], the postoperative accuracy of pedicle screw placements in our series was 313/317 (98.74%) in grade I, 3/317(0.95%) in grade II, 1/317(0.31%) in grade III, and 0/317 (0%) in grade IV.

## Discussion

### Accuracy of pedicle screw placement

Through use of the Renaissance robotic system, 98.74% of the K-wire placements deviated ≤3 mm from the planned trajectory. The accuracy in related studies has ranged from 85% to 99% [9, 14, 17]. Devito et al. reviewed 3271 pedicle screw placements in 635 patients from 14 medical centers and reported an accuracy of 98.3% [14]. Roser et al. reported an accuracy of 99% when the Renaissance robotic system was used [8].

Objective evidence from previous research suggests that compared with the accuracy of manual or image-guided operations, the accuracy of pedicle screw implantation is higher when the robotic system is used. Kantelhardt et al. compared pedicle screw placements performed manually with those in which the Renaissance robotic system was used and reported higher accuracy, lower radiation exposure, shorter hospital stays, and less pain killer usage in a group of patients who underwent robotic implantation [9]. Roser et al. compared manual, navigation-guided, and Renaissance robotic system-assisted pedicle screw placements and reported that the highest accuracy rate was achieved in the Renaissance robotic system group, the lowest radiation exposure was achieved in the navigation group, and the lowest operative time was achieved in the manual group. However, because the present study included only 10, 9, and 18 patients who underwent manual, navigation-guided, and robotic system-assisted pedicle screws placements, respectively, the efficiency and benefits of the robot-assisted pedicle screw placement required further evaluation through a larger series to confirm the findings [8].

Ringel et al. reported accuracies of 93% and 95% for manual and Renaissance robotic system–assisted pedicle screw placements, respectively. In addition, longer operative times were reported for when the Renaissance robotic system was used. They suggested that surgeons performing manual pedicle screw placements may require more experience than those using the Renaissance robotic system [17].

### Advantages of secondary registration

**Fig 4:**
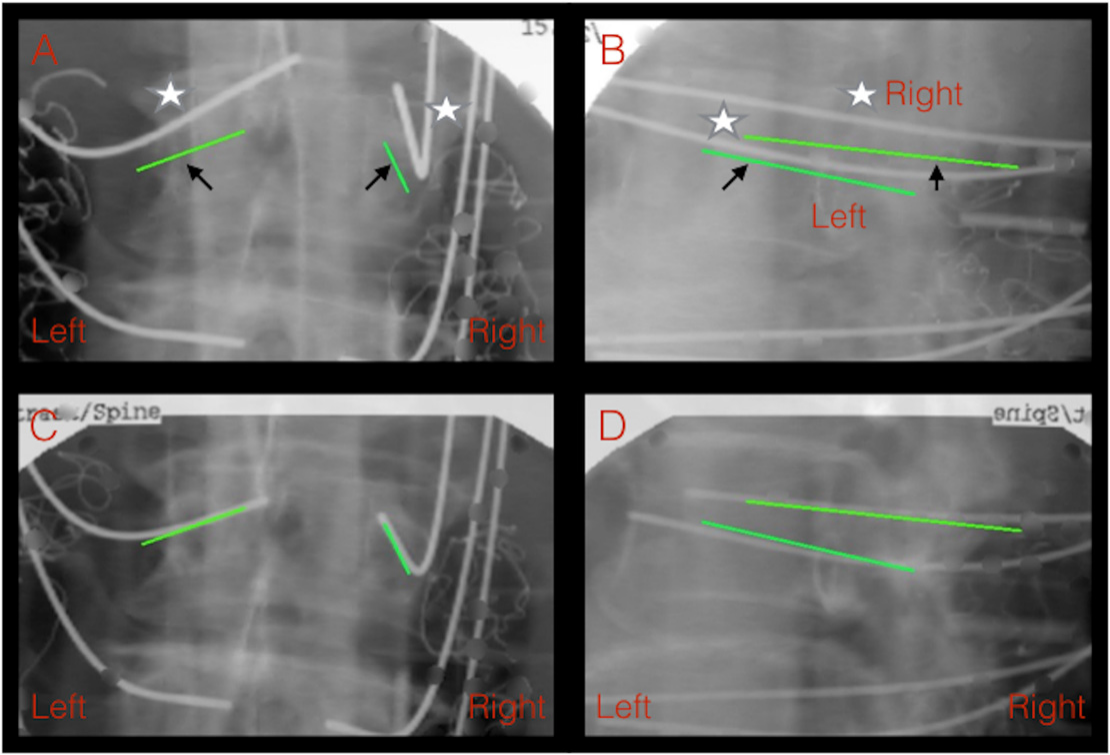
K-wire placement under the assistance of the robot before and after reregistration. A patient with lumbar spondylolisthesis received instrumentation from L3 to L5, and malpositioned bilateral L3 K-wires were noted initially. Fig 4A and 4B depict both left and right K-wire malpositions (Classification III) from the planned trajectory, as shown in the anteroposterior view (Fig 4A) and oblique view (Fig 4B). Fig 4C and 4D depict improved accuracy from classification type III to type I after the reregistration and adjustment of the K-wire placement.(Black arrow: planned trajectory; white star: implanted K-wire; green line: planned trajectory)

We proposed an intraoperative classification system based on relevant studies and categorized the accuracy status into three types: excellent, good, and malpositioned. According to our classification system, 94.00% and 98.74% of the robot-guided pedicle screw implantations in this study were in the excellent category before and after repositioning, respectively. However, the malpositioning rate was 5.99%; of these, 4.73% (type IIIa) were immediately repositioned using the robot system and 1.26% (type IIIb) were manually repositioned after a failed robot repositioning attempt. Intraoperative accuracy assessment through secondary registration can improve the accuracy of pedicle screw placement by providing more precise positioning and intraoperative evaluation, thus enabling surgeons to perform immediate repositioning (Fig 4). No secondary revision surgery was required in our study. Secondary registration provides several benefits such as the real-time realization of the occurrence of improper positioning, immediate evaluation of the requirement for repositioning, and instant analysis of the causes of improper screw placement. These characteristics provide possible management strategies for malpositioned screws.

### Factors influencing the accuracy of the robot-assisted system

Factors affecting the accuracy of the pedicle screw placements with the Renaissance robotic system include improper preoperative planning [13], unstable mounting [13, 18], poor registration quality [18], and poor drilling [8, 13, 17–19]. Poor drilling, such as skiving, is a major factor influencing the accuracy of pedicle screw placements. Irregular bony surfaces, steep entry point angles [13, 17–19], tissue pressure [14], high drilling pressure [19], and dull drill bits induce skiving. In the present study, most of the K-wire entry points deviated caudally and laterally. Most preoperatively planned entry points were located at the inferior articular surface of the facet joint. Ringel et al. [17] reported that the steeper the slope of the facet, the higher the possibility that the cannula will skid. Although the drill bit continues to show tactile movement, it results in frequent caudal and lateral skiving. Thus, the accuracy of pedicle screw placement can be reasonably improved by preventing skiving during drilling. For robot-guided drilling, we recommend careful preoperative planning and the use of soft tissue, low drill bit pressure, high-speed drilling, and sharp drill bits.

### Limitations

Although secondary registration offers an objective process for immediately repositioning K-wires, malpositions after transpedicular screw placement, K-wire bending, K-wire breakage, and even advertent advancement under the K-wire guidance may still occur. For K-wires deviating by >3 mm from the trajectory (type III classification), the K-wires may still be within the column of the pedicle without breaching the pedicle; regarding the K-wires in classified as type I or II, breaching of the pedicle may still occur. The K-wire should be closer to the trajectory because the robot offers an objective, steady, and safe means for performing transpedicular screw placements.

Second, because the accuracy of postoperative pedicle screws in this study was evaluated through postoperative biplanar conventional X-ray images rather than postoperative CT, the precision of the postoperative evaluation of transpedicular screw placements may be less accurate. Further research on the correlation between postoperative biplanar conventional X-ray and postoperative CT is warranted Regarding the factors affecting the accuracy of the robot-assisted system, skiving is believed to play a crucial role; however, other factors may also affect the accuracy. We could not conclusively determine the effect of skiving and thus additional studies evaluating these factors are warranted.

## Conclusion

Pedicle screw placement using the Renaissance robotic system offers high-accuracy pedicle screw placement, and secondary registration further improves the accuracy through obtaining a more precise positioning and intraoperative evaluation, enabling immediate repositioning. Furthermore, the K-wire tends to deviate caudally and laterally from the entry point, which is another characteristic of robot-assisted pedicle screw placement.

## Supporting Information

S1 File Clinical characteristics of patients receiving robotic-assisted transpedicle screws placement. S1 File details the patients database with clinical characteristics in this study, inclusive of patients’ characteristics, perioperative time, intra-operative X-ray exposure, classification system of secondary registration.
(XLS)
